# METTL14 Regulates PLAGL2/*β*-Catenin Signaling Axis to Promote the Development of Nonsmall Cell Lung Cancer

**DOI:** 10.1155/2023/4738586

**Published:** 2023-02-23

**Authors:** Qianhui Zhou, Xihua Lai, Yan Gao, Quefei Chen, Yuzhu Xu, Yi Liu

**Affiliations:** ^1^Department of Respiratory and Critical Care Medicine, Zhuzhou Central Hospital, Zhuzhou 412000, Hunan, China; ^2^Department of Cardiovascular Surgery, Zhuzhou Central Hospital, Zhuzhou 412000, Hunan, China

## Abstract

N6-methyladenosine (m6A) is an abundant eukaryotic mRNA modification involved in regulating the formation and metastasis of nonsmall cell lung cancer (NSCLC). We collected clinical NSCLC tissue and paracarcinoma tissue. Then methyltransferase-like 14 (METTL14), pleomorphic adenoma gene like-2 (PLAGL2), and *β*-catenin expressions were assessed using quantitative real-time PCR and western blot. PLAGL2, and *β*-catenin (nuclear) expressions were increased in NSCLC tissues. Cell proliferation, migration, invasion, and death were examined. PLAGL2 could activate *β*-catenin signaling to affect cell proliferation and migration abilities. RNA immunoprecipitation assay was operated to identify m6A modification levels of PLAGL2 after knockdown and overexpression of METTL14. PLAGL2 was regulated by METTL14-mediated m6A modification. Knockdown of METTL14 repressed cell proliferation, migration, and invasion, and promoted cell death. Interestingly, these effects were reversed when PLAGL2 was overexpressed. Finally, tumor formation in nude mice was performed to verify the role of the METTL14/PLAGL2/*β*-catenin signaling axis. Tumor formation in nude mice demonstrated METTL14/PLAGL2/*β*-catenin axis promoted NSCLC development *in vivo*. In brief, METTL14 promoted NSCLC development by increasing m6A methylation of PLAGL2 to activate *β*-catenin signaling. Our research provided essential clues for in-depth comprehension of the mechanism of NSCLC occurrence and development and also provided the basis for NSCLC treatment.

## 1. Introduction

Nonsmall cell lung cancer (NSCLC) accounts for 85% of all lung cancer incidence [1]. While it affects the quality of patients' life, it also increases the global economic burden. Due to a large number of mutations and general heterogeneity in this type of cancer, the use of traditional therapies has been challenging [2]. Small molecule tyrosine kinase inhibitors and immunotherapy have brought unprecedented survival benefits to selected patients. But overall cure rate and survival rate of NSCLC are still low [3]. At present, molecular-targeted therapy has also made great progress in the NSCLC treatment field [4]. Therefore, it is particularly essential to investigate the internal mechanism of NSCLC and provide potential scientific clues for subsequent treatment of NSCLC.

N6-methyladenosine (m6A) is an abundant eukaryotic mRNA modification and a common transcriptome modification in cancer. It was recently found to be involved in the regulation of NSCLC formation and metastasis [5]. Methyltransferase-like 14 (METTL14) is a core component of the m6A methyltransferase complex, and METTL14-induced abnormal m6A levels are related to tumorigenesis, proliferation, metastasis, and invasion [6]. METTL14 regulated various cancer occurrences and development, including breast cancer [7], colorectal cancer [8], and endometrioid epithelial ovarian cancer [9]. In NSCLC, knockdown of METTL14 inhibited Twist-mediated activation of AKT signaling to suppress NSCLC malignancy. This revealed METTL14 might be a potential therapeutic target for NSCLC [10]. As a writer of m6A methylation modification, METTL14 could promote the methylation modification of mRNA, thereby affecting its protein expression [11, 12]. Pleomorphic adenoma gene like-2 (PLAGL2) is the zinc finger protein transcription factor. It is overexpressed in many malignant tumors (gastric cancer, colorectal cancer, and breast cancer) and could facilitate tumor proliferation, migration, and invasion [13–15]. Overexpression of PLAGL2 has been implicated in lung cancer [16]. We predicted m6A modification of PLAGL2 and METTL14 acting on PLAGL2 by RMBase and m6A2Target. However, METTL14 and PLAGL2 mechanism in NSCLC remains to be investigated.

*β*-catenin is the complete structural component of cadherin-based and adherent junctions [17]. Abnormal *β*-catenin activating in nuclear is related to many human cancers [18]. Furthermore, METTL14 attenuated cardiac ischemia-reperfusion injury via activating Wnt/*β*-catenin in an m6A-dependent manner, which provided a new therapeutic target for ischemic heart disease [19]. In addition, related studies have shown PLAGL2 could regulate *β*-catenin and its downstream pathways and promote tumor genesis and development [14, 20]. However, there have been no studies of PLAGL2 with *β*-catenin in NSCLC.

In our research, we hypothesized METTL14 promoted PLAGL2 expression by increasing m6A methylation modification of PLAGL2, activating *β*-catenin signaling, thereby affecting NSCLC development. To this end, we collected clinical NSCLC tissue and paracarcinoma tissue for relevant testing. METTL14/PLAGL2/*β*-catenin role was also investigated *in vivo* and *in vitro* to investigate whether METTL14 regulated PLAGL2/*β*-catenin signaling axis to promote the development of NSCLC. Our research provided essential clues for an in-depth comprehension of the mechanism of NSCLC occurrence and development and also provided the basis for NSCLC treatment.

## 2. Materials and Methods

### 2.1. Collection of Clinical Samples

Tumor tissue and paracarcinoma tissue samples from NSCLC patients by iconography, serological or histopathological examination in Zhuzhou Central Hospital from November 2020 to August 2021 were collected. Clinical samples were divided into two groups: the NSCLC group (*n* = 5) and the paracarcinoma tissues group (*n* = 5). This study was approved by the Medical Ethics Committee of Zhuzhou Central Hospital (ZZCHEC2021092-01). The research was conducted according to the World Medical Association Declaration of Helsinki. All the information about the study will be fully explained to the subjects by the researchers. All the participants provided informed consent before sampling.

### 2.2. Cell Culture and Treatment

NSCLC cell A549 (ZQ0003) was obtained from Zhongqiao Xinzhou Biotech (Shanghai, China). It was cultured in DMEM high glucose medium with 10% FBS and 1% double antibodies. PLAGL2 and METTL14 were knocked down and overexpressed in A549 cells, and divided into NC (sh-PLAGL2 NC + oe-PLAGL2 NC), sh-PLAGL2 (sh-PLAGL2+ oe-PLAGL2 NC), and oe-PLAGL2 (sh-PLAGL2 NC + oe-PLAGL2) groups; NC (sh-METTL14 NC + oe-METTL14 NC), sh-METTL14 (sh-METTL14+ oe- METTL14 NC), and oe-METTL14 (sh-METTL14 NC + oe-METTL14) groups. To further study PLAGL2 and *β*-catenin function, we performed interference PLAGL2 and overexpression of *β*-catenin. The subgroups were sh-NC, sh-PLAGL2, sh-PLAGL2 + oe-NC, and sh-PLAGL2 + oe-*β*-catenin groups. Next, to verify METTL14 and PLAGL2's influence on NSCLC development, METTL14 was knocked down, and PLAGL2 was overexpressed. The subgroups were sh-NC, sh-METTL14, sh-METTL14 + oe-NC, and sh-METTL14 + oe-PLAGL2 groups. sh-PLAGL2, oe-PLAGL2, sh-METTL14, oe-METTL14, oe-*β*-catenin, and their control were provided in GenePharma (Shanghai, China). Cell transfection was performed according to Lipofectamine 3000 (L3000015, Invitrogen, USA) kit.

### 2.3. Hematoxylin-Eosin (HE) Staining

HE staining was performed to assess the pathological status of the tumor and paracarcinoma tissues [21]. The slices were placed in xylene. Each grade of ethanol was placed for 5 min. After dyeing with hematoxylin for 3 min, they were returned to blue with PBS. Eosin was dyed 5 s. Gradient alcohol (95–100%) was dehydrated. After taking them out, they were placed in xylene, sealed with neutral gum, and observed under the microscope (BA210T, Motic).

### 2.4. Quantitative Real-Time PCR (qRT-PCR)

In simple terms, total RNA was extracted through Trizol and reverse transcribed into cDNAs through cDNA reverse transcription kit (CW2569, CoWin Biosciences, Beijing, China). Ultra SYBR Mixture (CW2601, CoWin Biosciences, Beijing, China) was performed to evaluate the expression on ABI 7900 system. Using *β*-actin and U6 as reference genes, gene levels were calculated by 2^−ΔΔCt^. Primer sequences were as follows: METTL14-F: GTAGCACAGACGGGGACTTC, METTL14-R: TTGGTCCAACTGTGAGCCAG; PLAGL2-F: ACAATGCACCGCACAATGGC, PLAGL2-R: AAACAATCTCTTCACCGCGCTC;*β*-catenin-F: ATTCTTGGCTATTACGACAGACT,*β*-catenin-R: AGCAGACAGATAGCACCTT;*β*-actin-F: ACCCTGAAGTACCCCATCGAG,*β*-actin-R: AGCACAGCCTGGATAGCAAC.

### 2.5. Western Blot (WB)

RIPA kit (P0013B, Beyotime, Shanghai, China) extracted total protein from cells and tissues. Proteins were quantified according to the BCA protein assay kit (#7780, Cell Signaling Technology, USA) and isolated by 10% SDS-PAGE electrophoresis. Electrotransfer transferred the protein to the nitrocellulose filter membrane. The membrane was sealed with 5% skim milk to bind to nonspecific cells and incubated with primary antibody at 4°C overnight. For primary antibodies, METTL14 (26158-1-AP, 1 : 3000, Proteintech, USA), PLAGL2 (11540-1-AP, 1 : 300, Proteintech, USA), *β*-catenin (51067-2-AP, 1 : 1000, Proteintech, USA), PCNA (10205-2-AP, 1 : 1500, Proteintech, USA), and *β*-actin (60008-1-Ig, 1 : 5000, Proteintech, USA) were used. Then secondary antibodies were incubated. *β*-actin or PCNA acted as internal reference, and Odyssey Infrared Imaging System (Li-COR Biosciences, USA) assessed the protein bands.

### 2.6. RNA Immunoprecipitation (RIP) Assay

RIP lysis was first prepared. A549 cells were lysed with RIP lysis buffer and then incubated with RIP immunoprecipitation buffer containing magnetic beads coupled to antibodies against Ago2 (CST, USA) or anti-rabbit IgG (negative control, CST, USA). Precipitated RNA was evaluated by qRT-PCR. Total RNA was used as input controls. The primer sequences were PLAGL2-F: TCAGCTCCCTCCAAAATACCAG, PLAGL2-R: CTCCGCCAGAAAACTATCCAC.

### 2.7. Immunofluorescence (IF)

Cells were treated with slices, fixed with 4% paraformaldehyde. Slices were added 0.3% triton, permeated at 37°C. Slices were sealed with 5% BSA at 37°C. Cell slices were dropped with the appropriate dilution of primary antibody *β*-catenin (51067-2-AP, 1 : 100, Proteintech, USA). Then we added appropriate anti-rabbit-IgG-labeled fluorescent antibody and incubated them at 37°C for 90 min. DAPI working solution was nucleated for 10 min at 37°C, and then cells were sealed with buffer glycerol and observed under the fluorescence microscope.

### 2.8. Plate Clone Formation Assay

Cells were removed from each group at the exponential growth stage. Cells in each group were inoculated with 200 cells in each well in 6-well plates containing 1 mL culture medium restored to room temperature. Cells were placed in the 5% CO_2_, 37°C, and saturated humidity incubator for 2 to 3 weeks, during which the liquid was appropriately changed. We discarded the culture medium, carefully soaked PBS twice, and added 4% paraformaldehyde (1 mL) to fix the cells. We removed the fixing solution and added 1 mL of dye solution to the working solution to stain. After decolorization, the absorbance (OD) value at 550 nm was assessed by using the enzyme-plate reader.

### 2.9. Trypan Blue Staining

Cells were collected after digestion with trypsin and EDTA. We centrifuged collected cells at 1500 g for 1 min, discarded the supernatant, and resuspended cells with 1 mL or an appropriate cell suspension depending on the number of cells. Trypan blue staining was performed according to previous reports [22].

### 2.10. Wound Healing Assay

Cells were digested with trypsin to form single cell suspension and inoculated on 6-well culture plates (5 × 10^5^ cells per well). Cells were cultured in a 5% CO_2_ incubator at 37°C for 24 h until cells were covered with six-well plates. We scratched with a horizontal line perpendicular to the back of the six-hole plate. Serum-free DMEM medium (D5796, Sigma, USA) was added. Scratches at 0 h were photographed and taken under the inverted biological microscope (DSZ2000X, Cnmicro, China) after incubation for 24 and 48 h.

### 2.11. Transwell Assay

Cell invasion assays were applied in the Transwell chamber (3428, Corning, USA) with a matrix gel (354262, BD, USA). Cells were digested with trypsin into a single cell suspension and suspended in serum-free medium to 2 × 10^6^ cells/mL. The upper chamber was inoculated with 100 *μ*L cells, and the lower chamber was inoculated with 10% FBS complete medium (500 *μ*L). After being cultured in a 37°C incubator for 48 h, they were washed in the upper chamber with PBS. We fixed them with paraformaldehyde and removed the film. We dyed the film with crystal violet (0.1%) for 5 min. Upper outdoor surface cells were observed under the inverted microscope (DSZ2000X, Cnmicro, China). After decolorization by soaking in acetic acid, the number of cells was counted [23].

### 2.12. *In Vivo* Tumorigenesis

Twenty BALB/c nude mice (4–6 weeks) were randomly assigned to sh-NC, sh-METTL14, sh-METTL14+oe-NC, and sh-METTL14+oe-PLAGL2, with 5 mice in each group. Mice were raised under sterile conditions of ambient room temperature of 26–28°C, the humidity of 40–60%, and alternating day and night for 10 h/14 h. The Mice were fed sterile food and water. After 1 week of adaptive feeding, A549 cells that transfected with sh-NC, sh-METTL14, oe-NC, and oe-PLAGL2 were subcutaneously injected. The cell concentration was 5 × 10^6^/mL, and 200 *μ*L was injected [24, 25]. The formation of the transplanted tumor was observed, and tumors with a diameter ≥0.5 cm were considered tumor formation. Tumor volume was calculated. After the experiment was completed, we euthanized the mice under animal ethics. The mice were sacrificed with 150 mg/kg sodium pentobarbital.

### 2.13. Statistical Analysis

GraphPad Prism 8.0 software was applied for statistical analysis. Data were expressed as mean ± standard deviation (SD). First, normality and homogeneity of variance tests were performed. When the test conformed to the normal distribution and the variance was homogeneous, Student's *t*-test was used between the two groups. Comparisons among multiple groups were conducted by one-way analysis of variance (ANOVA), followed by Tukey's post hoc test. *P* < 0.05 indicated the difference was statistically significant.

## 3. Results

### 3.1. PLAGL2 Level Was Elevated in NSCLC Tissues

**Figure 1:**
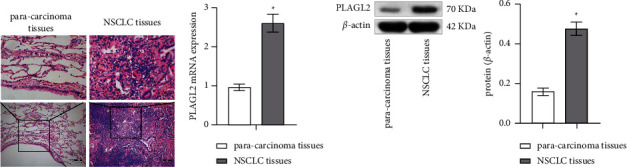
PLAGL2 level was elevated in NSCLC tissues. (a) HE staining assessed the pathological status. (b) PLAGL2 mRNA expression. (c) PLAGL2 protein expression. Student's *t*-test was used between the two groups. ^∗^*P* < 0.05 vs. paracarcinoma tissues.

First, HE staining assessed the pathological status. Compared with paracarcinoma tissues, NSCLC tissues had larger nuclei, larger nucleoplasmic ratio, more obvious histopathological atypia, and unclear tissue structure (Figure 1(a)). Subsequently, PLAGL2 expression was measured by qRT-PCR. Compared with paracarcinoma tissues, PLAGL2 expression was elevated in NSCLC tissues (Figure 1(b)). PLAGL2 expression was further verified by WB, and WB results were consistent with the trend of qRT-PCR (Figure 1(c)).

### 3.2. PLAGL2 Activated *β*-catenin Signaling to Affect Cell Proliferation and Migration

**Figure 2:**
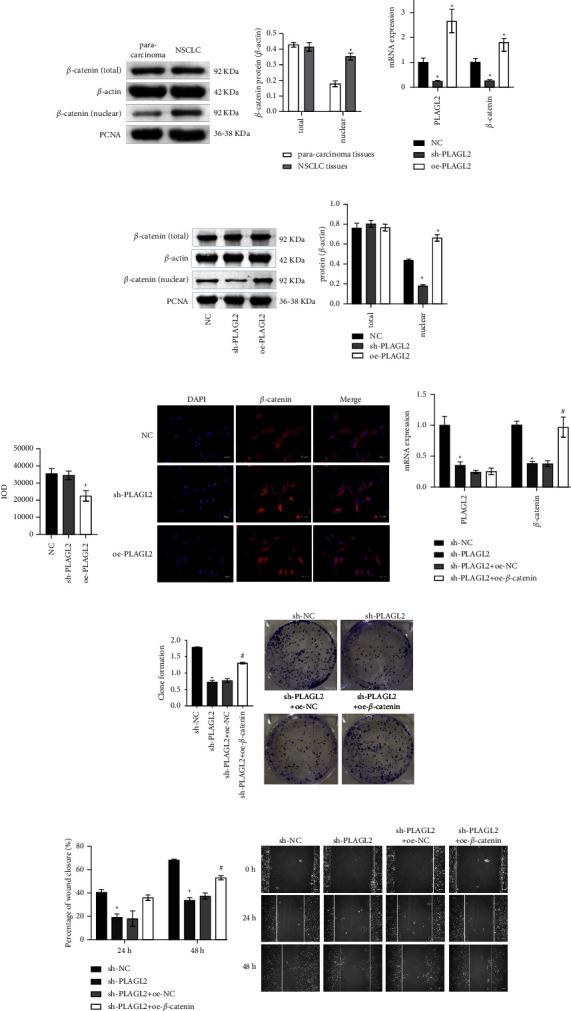
PLAGL2 activated *β*-catenin signaling to influence cell proliferation and migration. (a) *β*-catenin (total) and *β*-catenin (nuclear) protein expressions in NSCLC tissues. ^∗^*P* < 0.05 vs. paracarcinoma tissues. (b) PLAGL2 and *β*-catenin mRNA expressions. (c) *β*-catenin (total) and *β*-catenin (nuclear) protein expressions in cells. (d) IF detection of *β*-catenin localization. ^∗^*P* < 0.05 vs. NC. (e) PLAGL2 and *β*-catenin mRNA expressions. (f) Plate clone formation assay to evaluate cell proliferation. (g) Cell migration was tested by wound healing assay. Student's *t*-test was used between the two groups. Comparisons among multiple groups were conducted by one-way analysis of variance (ANOVA), followed by Tukey's post hoc test. ^∗^*P* < 0.05 vs sh-NC, ^#^*P* < 0.05 vs sh-PLAGL2 + oe-NC.

Through a literature search, we found PLAGL2 regulated *β*-catenin and its downstream pathways and promoted tumor genesis and development [14, 20]. Therefore, we wanted to investigate PLAGL2's role in *β*-catenin. First, *β*-catenin (total) and *β*-catenin (nuclear) expressions were evaluated by WB. *β*-catenin (nuclear) level was promoted in NSCLC tissues than paracarcinoma tissues, but *β*-catenin (total) was not significantly different (Figure 2(a)). Subsequently, PLAGL2 was knocked down and overexpressed in A549 cells. PLAGL2 level in the sh-PLAGL2 group was repressed, and that in the oe-PLAGL2 group was increased than the NC group, indicating that PLAGL2 was successfully knocked down and overexpressed. At the same time, *β*-catenin expression was repressed in the sh-PLAGL2 group and accelerated in the oe-PLAGL2 group compared with the NC group (Figure 2(b)). WB further verified that *β*-catenin (nuclear) expression was inhibited in the sh-PLAGL2 group and accelerated in the oe-PLAGL2 group compared with the NC group. *β*-catenin (total) showed no significant difference (Figure 2(c)). IF further demonstrated *β*-catenin location. Compared with the NC group, *β*-catenin in the oe-PLAGL2 group showed nuclear transfer (Figure 2(d)). Then we performed interference with PLAGL2 and overexpression of *β*-catenin. PLAGL2 level was repressed in sh-PLAGL2 group than sh-NC. *β*-catenin expression was elevated in the sh-PLAGL2 + oe-*β*-catenin group compared with the sh-PLAGL2 + oe-NC group, indicating that we successfully interfered with PLAGL2 and overexpressed *β*-catenin (Figure 2(e)). Plate clone formation and wound healing assays showed that the number of clones and migration were repressed in the sh-PLAGL2 group than the sh-NC group. After *β*-catenin was overexpressed, the number of clones and migration increased (Figures 2(f) and 2(g)). This suggested that PLAGL2 activated *β*-catenin signaling to influence cell proliferation and migration.

### 3.3. PLAGL2 Signaling Was Regulated by METTL14-Mediated m6A Modification

**Figure 3:**
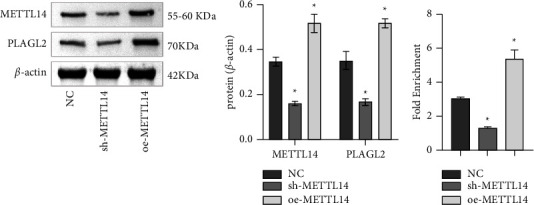
PLAGL2 signaling was regulated by METTL14-mediated m6A modification. (a) METTL14 and PLAGL2 expressions were detected by WB. (b) RIP identified m6A modification levels of PLAGL2 after knockdown and overexpression of METTL14. Comparisons among multiple groups were conducted by one-way analysis of variance (ANOVA), followed by Tukey's post hoc test. ^∗^*P* < 0.05 vs. NC.

First, we predicted m6A modification of PLAGL2 by RMBase and predicted METTL14 acted on PLAGL2 via m6A2Target. Therefore, we knocked down and overexpressed METTL14 to explore the mechanism of METTL14 and PLAGL2. METTL14 level in the sh-METTL14 group was suppressed and that in the oe-METTL14 group was increased than the NC group, indicating that METTL14 was successfully knocked down and overexpressed. In addition, PLAGL2 expression was repressed in the sh-METTL14 group, and accelerated in the oe-METTL14 group compared with the NC group (Figure 3(a)). Then we used RIP to identify the m6A modification of PLAGL2 after knockdown and overexpression of METTL14. The results revealed the m6A modification of PLAGL2 was repressed in the sh-METTL14 group and accelerated in the oe-METTL14 group than the NC group (Figure 3(b)). Collectively, PLAGL2 signaling was regulated by METTL14-mediated m6A modification.

### 3.4. METTL14 Regulated PLAGL2 Signaling to Affect NSCLC Cell Function

**Figure 4:**
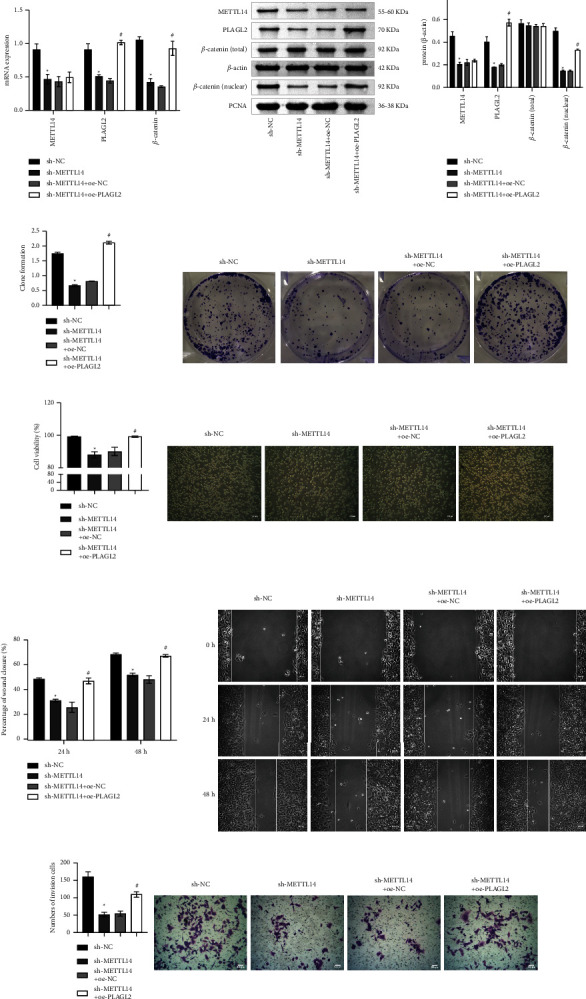
METTL14 regulated PLAGL2 signaling to affect NSCLC cell function. (a) METTL14, PLAGL2, and *β*-catenin mRNA expressions. (b) METTL14, PLAGL2, *β*-catenin (total), and *β*-catenin (nuclear) protein expressions. (c) Plate clone formation assay to measure cell proliferation. (d) Trypan blue staining to detect cell death (cell viability). (e) Cell migration was monitored by wound healing assay. (f) Cell invasion was determined by Transwell assay. Comparisons among multiple groups were conducted by one-way analysis of variance (ANOVA), followed by Tukey's post hoc test. ^∗^*P* < 0.05 vs. sh-NC, ^#^*P* < 0.05 vs. sh-METTL14 + oe-NC.

Next, to verify METTL14 and PLAGL2's influence on NSCLC development deeply, METTL14 was knocked down, and PLAGL2 was overexpressed. qRT-PCR showed METTL14 level was repressed in the sh-METTL14 than the sh-NC. In the sh-METTL14 + oe-PLAGL2 group, PLAGL2 expression was increased compared with the sh-METTL14 + oe-NC group. This indicated METTL14 was knocked down successfully, and PLAGL2 was successfully overexpressed (Figure 4(a)). WB showed decreased expressions of METTL14, PLAGL2, and *β*-catenin (nuclear) in the sh-METTL14 than in the sh-NC. PLAGL2 and *β*-catenin (nuclear) expressions were elevated in the sh-METTL14 + oe-PLAGL2 compared with the sh-METTL14 + oe-NC. *β*-catenin (total) showed no significant difference (Figure 4(b)). Cell function experiments showed knockdown of METTL14 reduced cell proliferation, migration, and invasion and facilitated cell death (cell viability decreased). After PLAGL2 was overexpressed, cell proliferation, migration, and invasion were increased, and cell death was suppressed (cell viability increased) (Figures 4(c)–4(f)).

### 3.5. METTL14/PLAGL2/*β*-Catenin Axis Promoted NSCLC Development *In Vivo*

**Figure 5:**
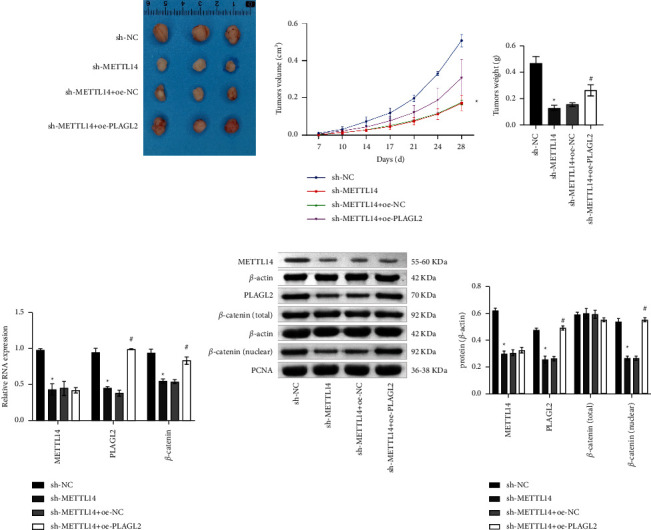
METTL14/PLAGL2/*β*-catenin axis promoted NSCLC development *in vivo*. (a) Tumor images and tumor volume. (b) Tumor weight. (c) METTL14, PLAGL2, and *β*-catenin mRNA expressions. (d) METTL14, PLAGL2, *β*-catenin (total), and *β*-catenin (nuclear) protein expressions. Comparisons among multiple groups were conducted by one-way analysis of variance (ANOVA), followed by Tukey's post hoc test. ^∗^*P* < 0.05 vs. sh-NC, ^#^*P* < 0.05 vs. sh-METTL14 + oe-NC.

To further validate METTL14/PLAGL2/*β*-catenin function, we performed *in vivo* experiments. Knockdown of METTL14 reduced tumor volume and weight. Tumor volume and weight increased after PLAGL2 was overexpressed (Figures 5(a) and 5(b)). Compared with sh-NC, METTL14 expression was decreased in sh-METTL14. In comparison with sh-METTL14 + oe-NC, PLAGL2 was elevated in sh-METTL14 + oe-PLAGL2, indicating METTL14 was successfully knocked down and PLAGL2 was successfully overexpressed. Besides, *β*-catenin expression was inhibited after the knockdown of METTL14. After PLAGL2 was overexpressed, *β*-catenin expression increased (Figure 5(c)). WB also showed decreased expressions of METTL14, PLAGL2, and *β*-catenin (nuclear) in sh-METTL14 compared to sh-NC. PLAGL2 and *β*-catenin (nuclear) expressions were elevated in sh-METTL14 + oe-PLAGL2 than sh-METTL14+oe-NC. *β*-catenin (total) showed no significant difference (Figure 5(d)). All in all, METTL14/PLAGL2/*β*-catenin axis promoted NSCLC development *in vivo*.

## 4. Discussion

NSCLC is one of the most devastating cancers, with high mortality worldwide [26]. New treatment options for NSCLC have become available through extensive in-depth genomic studies to improve preclinical disease patterns and identify specific toxicity of targeted therapies [27]. In our research, we collected clinical NSCLC and paracarcinoma tissues and investigated METTL14/PLAGL2/*β*-catenin's role *in vivo* and *in vitro*. Our results demonstrated METTL14 promoted NSCLC development by increasing m6A methylation of PLAGL2 to activate *β*-catenin signaling. There has been no reported research on the related mechanism of the METTL14/PLAGL2/*β*-catenin axis in NSCLC, which is also our innovation of study.

Researchers believed PLAGL2 was overexpressed in different malignant tumors, and it could facilitate tumor proliferation, migration, invasion, and self-renewal [28]. We also found that PLAGL2 expression was elevated in NSCLC tissues. Wu et al. reported PLAGL2 facilitated *β*-catenin expression and nuclear translocation by inhibiting *β*-catenin phosphorylation level [20]. Liu et al. showed that lncRNA HCP5 could activate Wnt/*β*-catenin/Cyclin D1 signal through PLAGL2 in multiple myeloma [29]. In addition, PLAGL2 may be a very upstream key molecule regulating epithelial-mesenchymal transition and participated in Wnt/*β*-catenin signaling in colorectal adenocarcinoma [30]. These studies suggest that PLAGL2 may play an important role in cancer by modulating *β*-catenin signaling. In this study, we revealed *β*-catenin (nuclear) level was elevated in NSCLC tissues than paracarcinoma tissues. Moreover, PLAGL2 activated *β*-catenin signaling to influence cell proliferation and migration, suggesting that PLAGL2 played an important role in NSCLC by regulating *β*-catenin signaling. This is also the first time we have reported the study of PLAGL2 and *β*-catenin signaling in NSCLC.

m6A is the enzyme that plays a vital role in mRNA splicing, translation, and stabilization [31]. It regulates biological metabolism, cell differentiation and cycle, and responses to heat shock stress and cancer [32]. METTL14 is a well-known RNA m6A that plays a vital role in tumor growth by controlling RNA work [33]. Previous studies have reported that METTL14 can increase m6A modification of pri-miR-19a and promote mature miR-19a processing, thereby facilitating atherosclerotic vascular endothelial cell proliferation and invasion [34]. m6A regulator METTL14 has been reported to be differentially expressed between TP53-mutant and wild-type NSCLC [35]. But the mechanism of METTL14-mediated m6A modification with PLAGL2 is still unclear. We found that PLAGL2 signaling was regulated by METTL14-mediated m6A modification. Furthermore, METTL14 regulated PLAGL2 signaling to affect NSCLC cell function. This is also our first report of METTL14-mediated m6A modification and PLAGL2 in NSCLC. Through *in vivo* experiments, we further validated METTL14/PLAGL2/*β*-catenin axis promoted NSCLC development.

However, our study has some limitations. To verify whether PLAGL2 signaling was regulated by METTL14-mediated m6A modification, we should first apply RIP to identify whether PLAGL2 was modified by m6A and then use RIP to identify the m6A modification of PLAGL2 after knockdown and overexpression of METTL14. However, due to time and financial constraints, we directly used RIP to identify the m6A modification of PLAGL2 after the knockdown and overexpression of METTL14. Although our results also confirmed that PLAGL2 signaling was regulated by METTL14-mediated m6A modification, the results need to be proved step by step. In the future, we will further verify whether PLAGL2 was modified by m6A. Furthermore, the mechanism of METTL14 and the m6A methylation modification of PLAGL2 *in vivo* and the signaling pathways involved need to be further explored.

In conclusion, we demonstrated increased PLAGL2 and *β*-catenin (nuclear) expressions in NSCLC tissues. Furthermore, we conducted a preliminary exploration of the mechanisms involved in METTL14/PLAGL2/*β*-catenin. We found METTL14/PLAGL2/*β*-catenin axis promoted NSCLC development *in vitro* and *in vivo* experiments. Our research provides important clues for an in-depth comprehension of the mechanism of NSCLC occurrence and development and also provides a reference for NSCLC treatment.

## Data Availability

The data used to support the findings of this study are available from the corresponding author upon request.
